# *trans*-Chlorido(dimethyl sulfoxide-κ*S*)(pyridine-2-carboxyl­ato-κ^2^ *N*,*O*)platinum(II)

**DOI:** 10.1107/S1600536810005520

**Published:** 2010-02-13

**Authors:** Kwang Ha

**Affiliations:** a School of Applied Chemical Engineering, The Research Institute of Catalysis, Chonnam National University, Gwangju 500-757, Republic of Korea

## Abstract

In the title complex, [Pt(C_6_H_4_NO_2_)Cl(C_2_H_6_OS)], the Pt^II^ ion is in a distorted square-planar environment defined by the N and O atoms from the chelating pyridine-2-carboxyl­ate (pic) anionic ligand, one S atom of the dimethyl sulfoxide mol­ecule and one Cl ion. The complex is disposed about a crystallographic mirror plane parallel to the *ac* plane passing through all the atoms of the complex except the methyl atoms of the dimethyl sulfoxide. The mol­ecules are stacked in columns along the *b* axis with a Pt⋯Pt distance of 4.9508 (5) Å. Within the column, inter­molecular C—H⋯O hydrogen bonds and weak π–π inter­actions between adjacent pyridine rings are present, the shortest centroid–centroid distance being 5.153 (4) Å.

## Related literature

For the crystal structure of the title complex with the monoclinic space group *P*2_1_/*n*, see: Annibale *et al.* (1986 ▶). For details of Pt(IV)–pic complexes, see: Griffith *et al.* (2005 ▶); Kim *et al.* (2009 ▶).
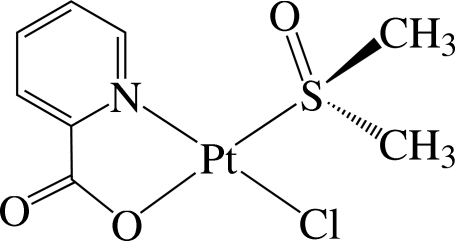


## Experimental

### 

#### Crystal data

[Pt(C_6_H_4_NO_2_)Cl(C_2_H_6_OS)]
*M* *_r_* = 430.77
Orthorhombic, 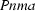

*a* = 19.5900 (15) Å
*b* = 6.9450 (6) Å
*c* = 8.1266 (6) Å
*V* = 1105.64 (15) Å^3^
*Z* = 4
Mo *K*α radiation
μ = 13.11 mm^−1^
*T* = 200 K
0.21 × 0.17 × 0.09 mm

#### Data collection

Bruker SMART 1000 CCD diffractometer
Absorption correction: multi-scan (*SADABS*; Bruker, 2000 ▶) *T*_min_ = 0.631, *T*_max_ = 1.000
6423 measured reflections
1169 independent reflections
1085 reflections with *I* > 2σ(*I*)
*R*_int_ = 0.042

#### Refinement

*R*[*F* ^2^ > 2σ(*F* ^2^)] = 0.027
*wR*(*F* ^2^) = 0.065
*S* = 1.10
1169 reflections
89 parameters
H-atom parameters constrained
Δρ_max_ = 2.60 e Å^−3^
Δρ_min_ = −0.79 e Å^−3^

### 

Data collection: *SMART* (Bruker, 2000 ▶); cell refinement: *SAINT* (Bruker, 2000 ▶); data reduction: *SAINT*; program(s) used to solve structure: *SHELXS97* (Sheldrick, 2008 ▶); program(s) used to refine structure: *SHELXL97* (Sheldrick, 2008 ▶); molecular graphics: *ORTEP-3* (Farrugia, 1997 ▶) and *PLATON* (Spek, 2009 ▶); software used to prepare material for publication: *SHELXL97*.

## Supplementary Material

Crystal structure: contains datablocks I. DOI: 10.1107/S1600536810005520/si2243sup1.cif

Structure factors: contains datablocks I. DOI: 10.1107/S1600536810005520/si2243Isup2.hkl

Additional supplementary materials:  crystallographic information; 3D view; checkCIF report

## Figures and Tables

**Table:** 

Pt1—O1	2.020 (5)
Pt1—N1	2.031 (7)
Pt1—S1	2.202 (2)
Pt1—Cl1	2.2945 (19)

**Table:** 

O1—Pt1—N1	81.0 (2)
O1—Pt1—S1	177.70 (16)
N1—Pt1—S1	101.31 (19)
O1—Pt1—Cl1	88.98 (16)
N1—Pt1—Cl1	169.97 (19)
S1—Pt1—Cl1	88.72 (7)

**Table 2:** Hydrogen-bond geometry (Å, °)

*D*—H⋯*A*	*D*—H	H⋯*A*	*D*⋯*A*	*D*—H⋯*A*
C1—H1⋯O3	0.95	2.16	2.995 (11)	145
C2—H2⋯O1^i^	0.95	2.35	3.255 (11)	158
C7—H7*A*⋯O2^ii^	0.98	2.42	3.323 (8)	152
C7—H7*B*⋯Cl1	0.98	2.77	3.355 (7)	119

Symmetry codes: (i) 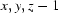; (ii) 
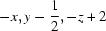
.

**Fig. 1.**
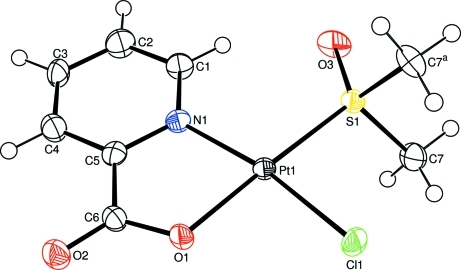
The structure of the title complex, with displacement ellipsoids drawn at the 50% probability level for non-H atoms [Symmetry code: (a) x, 1/2 - y, z].

**Fig. 2.**
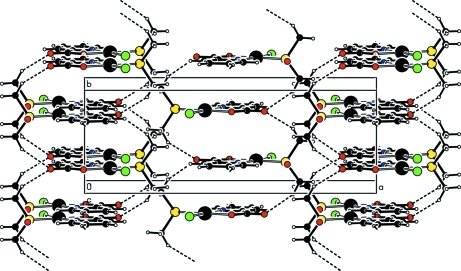
View of the unit-cell contents of the title complex. Hydrogen-bond interactions are drawn with dashed lines.

## supplementary crystallographic information

### Comment

The title complex, [Pt(C_6_H_4_NO_2_)Cl(C_2_H_6_OS)], crystallized in the orthorhombic space group *Pnma*, whereas, in the previously reported X-ray structure analysis, the complex crystallized in the monoclinic space group *P*2_1_/*n* (Annibale *et al.*, 1986). The Pt^II^ ion lies in a distorted square-planar environment defined by the N and O atoms from the chelating pyridine-2-carboxylate (pic) anionic ligand, one S atom of the dimethyl sulfoxide molecule and one Cl ion (Fig. 1). The tight O1—Pt1—N1 chelate angle [81.0 (2)°] results in non-linear trans axes [<O1—Pt1—S1 = 177.70 (16)° and <N1—Pt1—Cl1 = 169.97 (19)°] (Table 1). The complex is disposed about a crystallographic mirror plane parallel to the ac plane passing through all the atoms of the complex at the special positions (x,1/4,z), except the methyl atoms of the dimethyl sulfoxide (Fig. 2). The molecules are stacked in columns along the b axis with a Pt···Pt distance of 4.9508 (5) Å. In the column, intermolecular C—H···O hydrogen bond (Table 2) and weak π-π interactions between adjacent pyridine rings are present, the shortest centroid-centroid distance being 5.153 (4) Å, and the ring planes are parallel and shifted for 3.807 Å. The intramolecular C—H···O and C—H···Cl hydrogen bonds are also observed (Table 2).

### Experimental

Single crystals of the title complex were unexpectedly obtained by reacting K_2_PtCl_4_ (0.2000 g, 0.482 mmol) and pyridine-2-carboxylic acid (0.1192 g, 0.968 mmol) in H_2_O (10 ml) under reflux for 5 h. Crystals suitable for X-ray analysis were obtained by slow evaporation from a dimethyl sulfoxide solution of the pale yellow reaction product at 80 °C.

### Refinement

H atoms were positioned geometrically and allowed to ride on their respective parent atoms [C—H = 0.95 (aromatic) or 0.98 Å (CH_3_) and *U*_iso_(H) = 1.2*U*_eq_(C) or 1.5*U*_eq_(methyl C)]. The highest peak (2.60 e Å^-3^) and the deepest hole (-0.79 e Å^-3^) in the difference Fourier map are located 0.87 and 1.04 Å, respectively, from the atom Pt1.

### Crystal data

**Table:**

[Pt(C_6_H_4_NO_2_)Cl(C_2_H_6_OS)]	*F*(000) = 800
*M**_r_* = 430.77	*D*_x_ = 2.588 Mg m^−^^3^
Orthorhombic, *P**n**m**a*	Mo *K*α radiation, λ = 0.71073 Å
Hall symbol: -P 2ac 2n	Cell parameters from 3961 reflections
*a* = 19.5900 (15) Å	θ = 2.7–26.0°
*b* = 6.9450 (6) Å	µ = 13.11 mm^−^^1^
*c* = 8.1266 (6) Å	*T* = 200 K
*V* = 1105.64 (15) Å^3^	Block, colorless
*Z* = 4	0.21 × 0.17 × 0.09 mm

### Data collection

**Table:**

Bruker SMART 1000 CCD diffractometer	1169 independent reflections
Radiation source: fine-focus sealed tube	1085 reflections with *I* > 2σ(*I*)
graphite	*R*_int_ = 0.042
φ and ω scans	θ_max_ = 26.0°, θ_min_ = 2.7°
Absorption correction: multi-scan (*SADABS*; Bruker, 2000)	*h* = −24→23
*T*_min_ = 0.631, *T*_max_ = 1.000	*k* = −8→8
6423 measured reflections	*l* = −7→10

### Refinement

**Table:**

Refinement on *F*^2^	Primary atom site location: structure-invariant direct methods
Least-squares matrix: full	Secondary atom site location: difference Fourier map
*R*[*F*^2^ > 2σ(*F*^2^)] = 0.027	Hydrogen site location: inferred from neighbouring sites
*wR*(*F*^2^) = 0.065	H-atom parameters constrained
*S* = 1.10	*w* = 1/[σ^2^(*F*_o_^2^) + (0.0345*P*)^2^ + 1.8204*P*] where *P* = (*F*_o_^2^ + 2*F*_c_^2^)/3
1169 reflections	(Δ/σ)_max_ = 0.001
89 parameters	Δρ_max_ = 2.60 e Å^−^^3^
0 restraints	Δρ_min_ = −0.79 e Å^−^^3^

### Special details

**Table:**

Geometry. All esds (except the esd in the dihedral angle between two l.s. planes) are estimated using the full covariance matrix. The cell esds are taken into account individually in the estimation of esds in distances, angles and torsion angles; correlations between esds in cell parameters are only used when they are defined by crystal symmetry. An approximate (isotropic) treatment of cell esds is used for estimating esds involving l.s. planes.
Refinement. Refinement of *F*^2^ against ALL reflections. The weighted *R*-factor wR and goodness of fit *S* are based on *F*^2^, conventional *R*-factors *R* are based on *F*, with *F* set to zero for negative *F*^2^. The threshold expression of *F*^2^ > σ(*F*^2^) is used only for calculating *R*-factors(gt) etc. and is not relevant to the choice of reflections for refinement. *R*-factors based on *F*^2^ are statistically about twice as large as those based on *F*, and *R*- factors based on ALL data will be even larger.

### Fractional atomic coordinates and isotropic or equivalent isotropic displacement parameters (Å^2^)

**Table:**

	*x*	*y*	*z*	*U*_iso_*/*U*_eq_	
Pt1	0.087714 (15)	0.2500	1.04929 (4)	0.01771 (13)	
Cl1	0.14026 (11)	0.2500	1.3016 (2)	0.0265 (5)	
S1	0.18928 (11)	0.2500	0.9332 (2)	0.0209 (4)	
O1	−0.0036 (3)	0.2500	1.1647 (7)	0.0243 (13)	
O2	−0.1160 (3)	0.2500	1.1182 (8)	0.0311 (14)	
O3	0.1947 (3)	0.2500	0.7524 (8)	0.0353 (15)	
N1	0.0258 (3)	0.2500	0.8489 (8)	0.0204 (15)	
C1	0.0441 (5)	0.2500	0.6884 (10)	0.029 (2)	
H1	0.0911	0.2500	0.6596	0.035*	
C2	−0.0051 (5)	0.2500	0.5652 (11)	0.035 (2)	
H2	0.0085	0.2500	0.4530	0.042*	
C3	−0.0726 (4)	0.2500	0.6045 (12)	0.0274 (19)	
H3	−0.1066	0.2500	0.5211	0.033*	
C4	−0.0903 (4)	0.2500	0.7672 (12)	0.028 (2)	
H4	−0.1372	0.2500	0.7968	0.034*	
C5	−0.0410 (4)	0.2500	0.8904 (11)	0.0205 (17)	
C6	−0.0570 (4)	0.2500	1.0681 (10)	0.0217 (18)	
C7	0.2372 (3)	0.0507 (9)	1.0071 (8)	0.0300 (14)	
H7A	0.2145	−0.0694	0.9755	0.045*	
H7B	0.2405	0.0577	1.1273	0.045*	
H7C	0.2832	0.0539	0.9593	0.045*	

### Atomic displacement parameters (Å^2^)

**Table:**

	*U*^11^	*U*^22^	*U*^33^	*U*^12^	*U*^13^	*U*^23^
Pt1	0.0175 (2)	0.02225 (19)	0.0134 (2)	0.000	0.00107 (12)	0.000
Cl1	0.0231 (11)	0.0425 (12)	0.0138 (10)	0.000	−0.0040 (8)	0.000
S1	0.0195 (11)	0.0258 (10)	0.0174 (10)	0.000	0.0024 (8)	0.000
O1	0.018 (3)	0.044 (3)	0.011 (3)	0.000	0.003 (2)	0.000
O2	0.024 (3)	0.047 (4)	0.022 (3)	0.000	0.003 (3)	0.000
O3	0.036 (4)	0.055 (4)	0.015 (3)	0.000	0.016 (3)	0.000
N1	0.023 (4)	0.019 (3)	0.019 (4)	0.000	0.000 (3)	0.000
C1	0.028 (5)	0.049 (5)	0.009 (4)	0.000	0.003 (3)	0.000
C2	0.037 (6)	0.047 (6)	0.020 (5)	0.000	−0.001 (4)	0.000
C3	0.021 (5)	0.038 (5)	0.023 (5)	0.000	−0.006 (4)	0.000
C4	0.023 (5)	0.033 (5)	0.028 (5)	0.000	0.001 (4)	0.000
C5	0.023 (4)	0.013 (3)	0.025 (4)	0.000	−0.002 (4)	0.000
C6	0.019 (4)	0.024 (4)	0.022 (5)	0.000	0.002 (3)	0.000
C7	0.023 (3)	0.029 (3)	0.038 (4)	0.007 (3)	0.005 (3)	0.005 (3)

### Geometric parameters (Å, °)

**Table:**

Pt1—O1	2.020 (5)	C1—H1	0.9500
Pt1—N1	2.031 (7)	C2—C3	1.361 (13)
Pt1—S1	2.202 (2)	C2—H2	0.9500
Pt1—Cl1	2.2945 (19)	C3—C4	1.367 (14)
S1—O3	1.473 (6)	C3—H3	0.9500
S1—C7^i^	1.778 (6)	C4—C5	1.390 (13)
S1—C7	1.778 (6)	C4—H4	0.9500
O1—C6	1.308 (10)	C5—C6	1.477 (12)
O2—C6	1.225 (11)	C7—H7A	0.9800
N1—C5	1.352 (10)	C7—H7B	0.9800
N1—C1	1.352 (11)	C7—H7C	0.9800
C1—C2	1.390 (13)		
			
O1—Pt1—N1	81.0 (2)	C3—C2—H2	119.8
O1—Pt1—S1	177.70 (16)	C1—C2—H2	119.8
N1—Pt1—S1	101.31 (19)	C2—C3—C4	118.2 (8)
O1—Pt1—Cl1	88.98 (16)	C2—C3—H3	120.9
N1—Pt1—Cl1	169.97 (19)	C4—C3—H3	120.9
S1—Pt1—Cl1	88.72 (7)	C3—C4—C5	121.4 (8)
O3—S1—C7^i^	107.4 (3)	C3—C4—H4	119.3
O3—S1—C7	107.4 (3)	C5—C4—H4	119.3
C7^i^—S1—C7	102.3 (5)	N1—C5—C4	119.5 (8)
O3—S1—Pt1	119.5 (3)	N1—C5—C6	116.7 (7)
C7^i^—S1—Pt1	109.4 (2)	C4—C5—C6	123.8 (8)
C7—S1—Pt1	109.4 (2)	O2—C6—O1	123.7 (8)
C6—O1—Pt1	115.4 (5)	O2—C6—C5	121.7 (8)
C5—N1—C1	119.8 (7)	O1—C6—C5	114.7 (7)
C5—N1—Pt1	112.2 (6)	S1—C7—H7A	109.5
C1—N1—Pt1	127.9 (6)	S1—C7—H7B	109.5
N1—C1—C2	120.7 (8)	H7A—C7—H7B	109.5
N1—C1—H1	119.6	S1—C7—H7C	109.5
C2—C1—H1	119.6	H7A—C7—H7C	109.5
C3—C2—C1	120.3 (9)	H7B—C7—H7C	109.5
			
N1—Pt1—S1—O3	0.0	N1—C1—C2—C3	0.000 (2)
Cl1—Pt1—S1—O3	180.0	C1—C2—C3—C4	0.000 (2)
N1—Pt1—S1—C7^i^	124.3 (3)	C2—C3—C4—C5	0.000 (2)
Cl1—Pt1—S1—C7^i^	−55.7 (3)	C1—N1—C5—C4	0.000 (2)
N1—Pt1—S1—C7	−124.3 (3)	Pt1—N1—C5—C4	180.000 (2)
Cl1—Pt1—S1—C7	55.7 (3)	C1—N1—C5—C6	180.000 (2)
N1—Pt1—O1—C6	0.000 (2)	Pt1—N1—C5—C6	0.000 (2)
Cl1—Pt1—O1—C6	180.000 (2)	C3—C4—C5—N1	0.000 (2)
O1—Pt1—N1—C5	0.000 (1)	C3—C4—C5—C6	180.000 (2)
S1—Pt1—N1—C5	180.000 (1)	Pt1—O1—C6—O2	180.000 (2)
Cl1—Pt1—N1—C5	0.000 (4)	Pt1—O1—C6—C5	0.000 (2)
O1—Pt1—N1—C1	180.000 (1)	N1—C5—C6—O2	180.000 (2)
S1—Pt1—N1—C1	0.000 (1)	C4—C5—C6—O2	0.000 (2)
Cl1—Pt1—N1—C1	180.000 (3)	N1—C5—C6—O1	0.000 (2)
C5—N1—C1—C2	0.000 (2)	C4—C5—C6—O1	180.000 (2)
Pt1—N1—C1—C2	180.000 (1)		

Symmetry codes: (i) *x*, −*y*+1/2, *z*.

### Hydrogen-bond geometry (Å, °)

**Table:**

*D*—H···*A*	*D*—H	H···*A*	*D*···*A*	*D*—H···*A*
C1—H1···O3	0.95	2.16	2.995 (11)	145
C2—H2···O1^ii^	0.95	2.35	3.255 (11)	158
C7—H7A···O2^iii^	0.98	2.42	3.323 (8)	152
C7—H7B···Cl1	0.98	2.77	3.355 (7)	119

Symmetry codes: (ii) *x*, *y*, *z*−1; (iii) −*x*, *y*−1/2, −*z*+2.
